# Bessel beams with spatial oscillating polarization

**DOI:** 10.1038/srep30765

**Published:** 2016-08-04

**Authors:** Shiyao Fu, Shikun Zhang, Chunqing Gao

**Affiliations:** 1 School of Opto-Electronics, Beijing Institute of Technology, Beijing 100081, China

## Abstract

Bessel beams are widely used in optical metrology mainly because of their large Rayleigh range (focal length). Radial/azimuthal polarization of such beams is of interest in the fields of material processing, plasma absorption or communication. In this paper an experimental set-up is presented, which generates a Bessel-type vector beam with a spatial polarization, oscillating along the optical axis, when propagating in free space. A first holographic axicon (HA) HA1 produces a normal, linearly polarized Bessel beam, which by a second HA2 is converted into the spatial oscillating polarized beam. The theory is briefly discussed, the set-up and the experimental results are presented in detail.

Beams with a transverse homogeneous polarization, such as linearly, elliptically, or circularly polarized beams, are widely used in optics. In contrast to homogeneously polarized beams (circular, linear), vector vortex beams have a transversely varying polarization^1^, which can be described by the higher-order Poincaré sphere^2^,^3^, are attracting more and more attention for their unique characteristics. The two typical examples of vector beams are the radial or azimuthal polarization. Usually, these fields are eigen solutions of the vectorial Helmholtz equation and remain constant when propagating in free space. The transformation of the polarization states of vector beams requires wave plates, which increases the complexity of the set-up. In this paper a new kind of vector beams is presented named oscillating polarized (OP) vector beams. When propagating the polarization state is varying periodically with an oscillation length *z*_*t*_ determined by the two holographic axicons. The theory is briefly discussed, and an approach how to realize these beams experimentally is presented for different polarization orders. This simple set-up will be of interest for many applications, which require spatial polarization control.

The discovery of vector beams changed the understanding of polarization considerably, and has lead to an improvement of optical systems. For instance, higher absorption of a vector beam contributes to their application in laser plasma heating^4^, and radial polarized vector beams have favorable focusing property^5^. In addition, vector beams are also used in fields such as optical communications^6^,^7^,^8^, particle trapping^9^,^10^, surface plasma excitation^11^, image encryption^12^ and so on. In contrast to homogeneously polarized beams, vector beams have a unique spatial polarization structure. The field of such a beam reads:


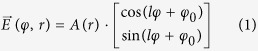


In Eq. (1), *A*(*r*) is the amplitude distribution, *φ* the azimuthal angle, *φ*_0_ the initial orientation of the field vector for *φ* *=* 0. *l* denotes the polarization order of the vector beam, also known as the topological charge of a vortex beam. Eq. (1) also represents a linearly polarized beam for *l* = 0 particularly. The state of polarization of a vector beam depends on the value of *φ*_0_. For instance, in the case of *l* = 1, *φ*_0_ = 0 is the radial polarization state and *φ*_0_ = π/2 is the azimuthal polarization state.

Vector beams can be generated by very different methods as inserting mode-selection elements in the laser resonator^13^,^14^,^15^, transformation from optical vortices outside the resonator^16^,^17^,^18^,^19^,^20^,^21^,^22^ and so on. However, the polarization state of vector beams stays constant along the optical axis and can’t be changed unless a wave plate is inserted in the optical path. The methods mentioned above may bring limitations for the application of vector beams. For example, in laser manufacturing, radially polarized beams are used for laser cutting^23^ and azimuthal polarized beams for punching^24^. If a vector beam contains these two polarization states in different propagation distance, the cutting and punching can be realized simultaneously. Moreno *et al*. have introduced an approach to generate nondiffracting Bessel beams with varying polarization states when propagating^25^. Nonetheless, the polarization in different positions of the optical axis is homogeneous.

In this paper, by using axicon holograms realized by spatial light modulators (SLM 1, SLM 2), a new method is presented to generate Bessel-type vector beams with spatial oscillating polarization along the *z*-axis. The state of polarization at different positions was measured with a polarizer. The experimental results fit well the theory.

## Results

### Principles of generating OP vector beams

Bessel beams, solutions of Helmholtz equation in cylindrical coordinates, are widely known as a non-diffraction or self-reconstructing light beam, which can reconstruct its electric field after passing through an obstruction^26^,^27^,^28^,^29^. Researches have done a lot in studying Bessel beams, and found their existence for atoms^30^ and electron waves^31^,^32^,^33^. The amplitude of a Bessel beam reads with Eq. (2):


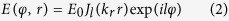


where *J*_*l*_ is the *l*-th order Bessel function, *k*_*r*_ is the radial wavenumber and *l* is the topological charge^26^,^34^,^35^. The term exp(*ilφ*) also means that the Bessel beam represents an optical vortex, which carries orbital angular momentum (OAM)^36^.

As a kind of Bessel-type vector beams^37^,^38^,^39^,^40^, OP vector beams can be generated from Bessel modes. Right and left circularly polarized Bessel beams, which have opposite topological charge can be combined and result in a Bessel-type vector beam, as shown in Eq. (3)


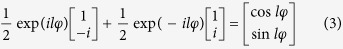


In Eq. (3), once an additional phase *ϕ*(*r*) is introduced in the left-circularly polarized component, and meanwhile it doesn’t change the phase of the right-circularly polarized state. Then Eq. (3) can be written as:


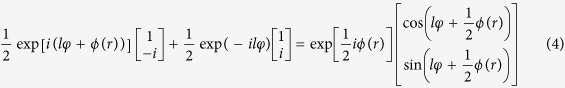


**Figure 1:**
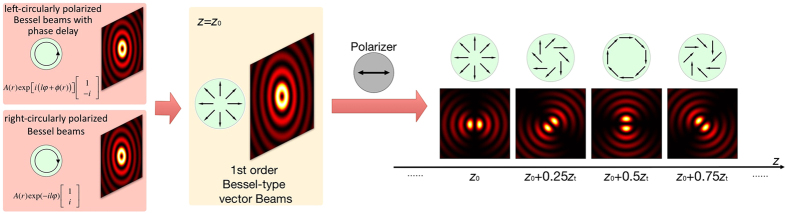
Schematic set-up to generate OP beams. The key idea is to introduce different additional phase-shifts at different positions of the axis. Then the state of polarization is varying with the propagation distance.

The introduction of *ϕ*(*r*) in the left-circularly polarized Bessel beam leads to a change of the initial polarization. For example, in the case of *l* = 1, the spatial polarization state will be changed from radial to azimuthal, when *ϕ*(*r*) varies between 0 and π. This effect can be used to generate OP vector beams as shown in Fig. 1.

**Figure 2:**
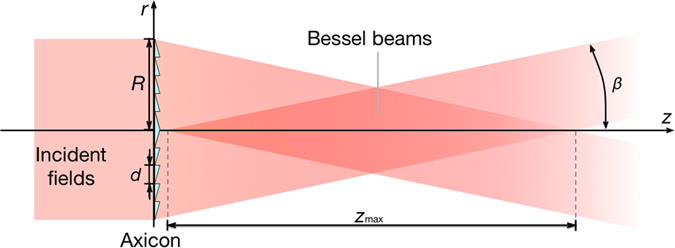
Axicons or axicon-holograms can be used to generate Bessel beams in the overlap region.

**Figure 3:**
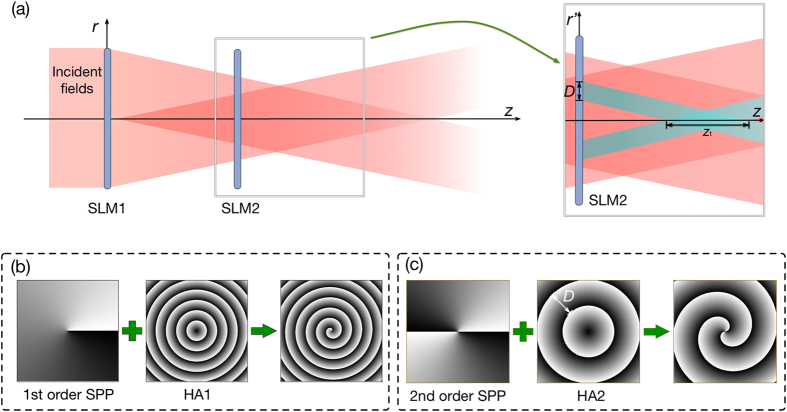
Two holographic axicons can introduce additional phase shifts along the optical path. (**a**) Scheme of realizing the additional phase in different propagation distance. SLM1 upload the combined hologram in (**b**) and SLM2 upload the combined hologram in (**c**). (**b,c**) show the hologram to generate first order OP vector beams. (**b**) The hologram on SLM1 to generate Bessel beams, which corresponds to an axicon (HA1) and a first order spiral phase plate (SPP). (**c**) The hologram on SLM2 to generate the additional phase shift, where HA2 combined with a second order SPP.

Various additional phase shifts in different propagation distances are generated by a special holographic axicon. An axicon is a tapered optical element with circular symmetry. Figure 2 illustrates the generation of Bessel beams by an axicon. When a plane wavefront passes an axicon, it will be transformed into a conical wave. In the overlap region Bessel-like waves are generated^41^. The maximum range of the Bessel beams is given by the length *z*_max_ of this region and reads^25^:


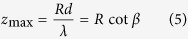


with *λ* the wavelength, *R* the radius of the axicon. If the beam size is smaller than the axicon’s base, *R* denotes the radius of the incident field. *β* is the refraction angle and *d* is the radial period of the axicon. If the incident field is horizontal linearly polarized, the Bessel beams will have the same polarization. In this experiment the axicon is replaced by a holographic axicon (HA) HA1, which can be uploaded on SLM, to generate linearly polarized Bessel beams. Now another holographic axicon HA2 with different period *D* is inserted and produces an additional phase shift depending now on the propagation distance *z* as shown in Fig. 3. Circularly polarized beams with different phase delay at different positions *z* are obtained after propagating through a 45° arranged quarter wave plate (QWP). The HA2’s phase distribution *ϕ*(*r*) is given by:


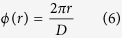


with *D* the period of the HA2. Eq. (5) with Eq. (6) results in


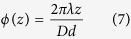


This is the additional phase shift of the propagating beam, which produces the oscillating states of polarization. The distance *z*_*t*_ between two equal states of polarization requires *ϕ*(*z*_*t*_) = 2π and delivers:


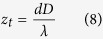


The overlap region of the additional phase shift must be smaller than the Bessel beam region which requires *z*_max_ > *z*_*t*_ or *D* < *R*.

### Experimental Setup

**Figure 4:**
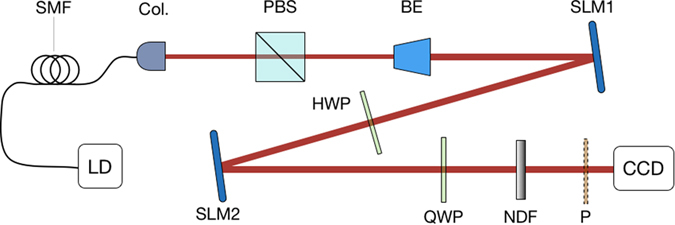
Experimental setup for generating OP vector beams. LD, laser diode. SMF, single mode fiber. Col., collimator. PBS, polarizing beam splitter. BE, beam expander. SLM1 and SLM2, liquid crystal spatial light modulators. HWP, half wave plate. QWP, quarter wave plate. NDF, neutral density filter. P, polarizer. CCD, infrared CCD camera.

The experimental setup for generating OP vector beams is shown in Fig.4. The fundamental Gaussian mode with a wavelength of 1550 nm is generated by a laser diode (LD) and coupled into a single mode fiber. The Gaussian output beam is collimated (Col.) with a diameter of 3 mm and polarized linearly horizontal by a beam splitter (PBS). Only for this polarization a pure phase modulation by the SLM is possible. The beam expander (BE), a concave lens *f* =  −50 mm and a convex lens *f* = 100 mm, enlarge the diameter to 6 mm.

The SLMs’ diffraction efficiency is 80%, nominal resolution 1920 × 1080 pixels, active area 15.36 mm × 8.64 mm and the pixel pitch 8.0 μm (Holoeye, PLUTO-TELCO-013-C). Utilizing the performance of the polarization control^42^ and the phase modulation of SLM, we can use two SLMs to accomplish all the calculation process discussed in section 2. For generating Bessel beams, we upload the hologram of an axicon (HA1) combined with *l*-th order spiral phase plate (SPP), which is shown in Fig. 3(b), on SLM1. The generated horizontal linearly polarized Bessel beam is of −*l*-th order, because of the reflective SLM 1. The reflection added by SLM1 contributes to the opposite topological charge. The half wave plate (HWP) is placed at an angle of 22.5° to the horizontal plane in order to rotate the polarization orientation of the Bessel beam at 45°. Thus, the generated Bessel beam has two linearly polarized components with equal intensity, a horizontal and a vertical one. After reflected by SLM2, only the horizontal polarization component will be modulated, due to the polarization feature of the SLM 1. The hologram uploaded on SLM2 consists of HA2 and a 2*l*-th order SPP, which introduces an additional phase *ϕ*(*r*) of the horizontal linearly polarized Bessel beam, as shown in Fig. 3(c). The topological charge of the horizontal component of the Bessel beam incident on SLM2 can be expressed as −(−*l* + 2*l*) = −*l*, which means it will not be changed. The topological charge of the vertical component will be opposite, for it is reflected by SLM2 once and is not modulated. Therefore, the light reflected by SLM2 consists of a horizontal and a vertical linearly polarized Bessel beam with opposite topological charges. The horizontal polarized component has a phase delay. A 45° quarter-wave plate (QWP) is used to transform the two combined linearly polarized Bessel beams into left and right circularly polarized helical beams. Eq. (4) is satisfied and an OP vector beam is generated.

A rotated polarizer is used to check the polarization distribution of the OP vector beam. An infrared CCD camera with the spectral range of 900 nm~1700 nm is used for the detection.

**Figure 5:**
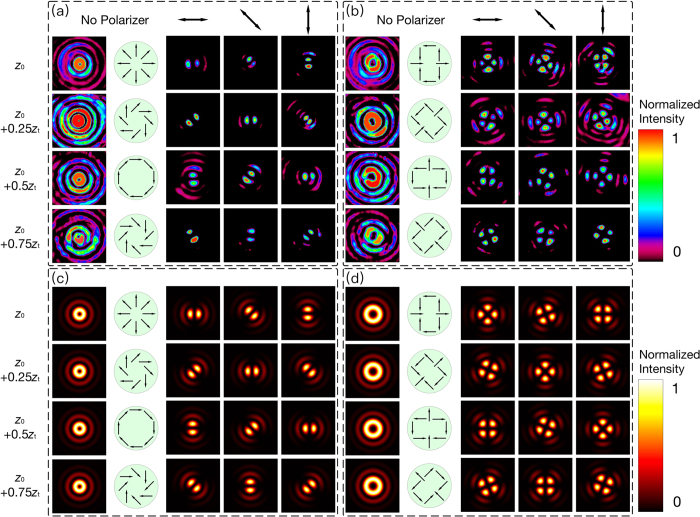
Experimental and simulated results of generating 1^st^ and 2^nd^ order OP vector beams. (**a**) Experimental 1^st^ order OP vector beams. (**b**) Experimental 2^nd^ order OP vector beams. (**c**) Simulated 1^st^ order OP vector beams. (**d**) Simulated 2^nd^ order OP vector beams. From left to right, the observed patterns without and with polarizer at 0°, 45°, 90°, respectively. From top to bottom, the patterns observed by CCD camera at different positions on the optical axis. The green areas show the spatial distribution of the polarization at different positions.

As previously mentioned, the pixel pitch of the SLMs is 8.0 μm. For this reason, the radial period of the HA1 *d* and the period of the HA2 *D* should be set as multiples of 8 when designing the hologram shown in Fig. 5. Only by this way each liquid crystal of the SLM can be well encoded. The period of the HA1 was 880 μm, and the period of HA2 was 2184 μm. Then the spatial period of the polarization becomes with Eq. (8) *z*_*t*_ = 1.24 m.

The radius of the incident beam with *R* = 3 mm is smaller than the axicon’s base and Eq. (5) delivers for the maximum range *z*_max_ = 1.70 m. The distance between SLM1 and SLM2 is 0.43 m, the free moving range for the CCD camera behind SLM2 is 1.27 m.

### Experimental results

Figure. 5 summarizes the experimental and simulated results of 1^st^ and 2^nd^ order OP vector beams. One can see that the experimental pattern fit well with theory. The intensity distribution of the *l*-th order beam is similar to a Bessel beam whose topological charge is ±*l*. The patterns are divided into 2*l* main lobes after passing through a polarizer. In the same location, for instance, *z* = *z*_0_, the lobes will rotate at an angle of *θ*/*l* when the polarizer rotates at an angle of *θ*. The absence of the side lobes in some of the patterns is caused by the lower transmittance of the NDF, to make the main lobes more clearly. The unique characteristic of the OP beams is the linear rotation of the polarization distribution along the optical axis. When moving the CCD camera along the axis over a distance of 0.25*z*_*t*_, the lobes will rotate by an angle of *π*/(4*l*), which can be explained by Eq. (7) and Eq. (4). Eq. (7) can be written as:


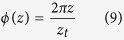


which delivers *ϕ* *=* *π*/2 if *z* = 0.25*z*_*t*_. It is clear from Eq. (3) that the rotation angle of the patterns behind a polarizer is *ϕ*/2*l*. Hence, the rotation angle of *π*/(4*l*) is obtained.

In order to verify quantitatively this variation, the rotation angle was measured along the *z*-axis with a moving CCD-camera. In the measurement, the polarizer is placed at the angle of 0°. In this case, the rotation angle *θ* should be a linear function of the camera’s position, which can be written as:


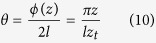


**Figure 6:**
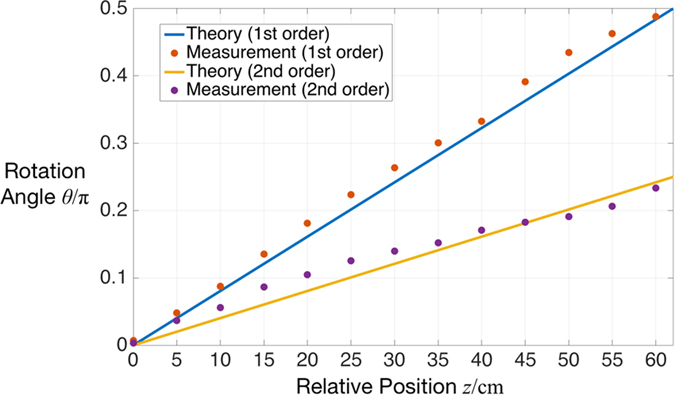
The angle of rotation vs the z-position for 1^st^ and 2^nd^ order OP vector beams behind a 0°- polarizer.

The pattern were measured in steps of Δ*z* = 0.05 m. After the image processing of each pattern, the corresponding relationship between the rotation angle and CCD’s position is obtained. The measurement was performed for the 1^st^ order and the 2^nd^ order OP vector beams. The results are shown in Fig. 6 and confirm well the theoretical considerations.

## Discussion

In this paper, we demonstrate experimentally and theoretically a new kind of Bessel beams (OP vector beams) with spatial oscillating, only by applying a polarization control phase-plates. In the experiment, two different holograms are uploaded on the two SLMs. The 1^st^ and the 2^nd^ order OP-vector beams were generated in the experiment. In addition, the linear variation of the spatial polarization when propagating is illustrated.

The OP vector beams exist in the Bessel overlap region only, which is also called the nondiffraction zone. It can be extended by enlarging the radial period of the axicon (HA1). Another important feature of OP vector beams is the spatial variation period, which is related to the radial period of HA1 and HA2, and the wavelength of the incident beams. Arbitrary variation periods can be designed by proper choice of the parameters.

## Methods

### Measurement of the rotation angle

In order to verify the rotation angle of the patterns, the center of gravity of each lobe in the optical fields has to be measured. It is given by the first intensity moment^43^:


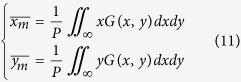


where *G*(*x*, *y*)∈[0, 1] is the normalized gray level in the position (*x*, *y*). *P* is the sum of all local gray levels:


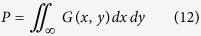


There are two lobes in the case of 1^st^ order OP-vector beams. Therefore, the rotation angle can be calculated by:


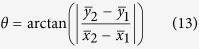


As for the 2^nd^ order OP vector beams, two of the four lobes in the diagonal were analyzed. The rotation angle is given by:


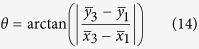


## Additional Information

**How to cite this article**: Fu, S. *et al*. Bessel beams with spatial oscillating polarization. *Sci. Rep.* **6**, 30765; doi: 10.1038/srep30765 (2016).
